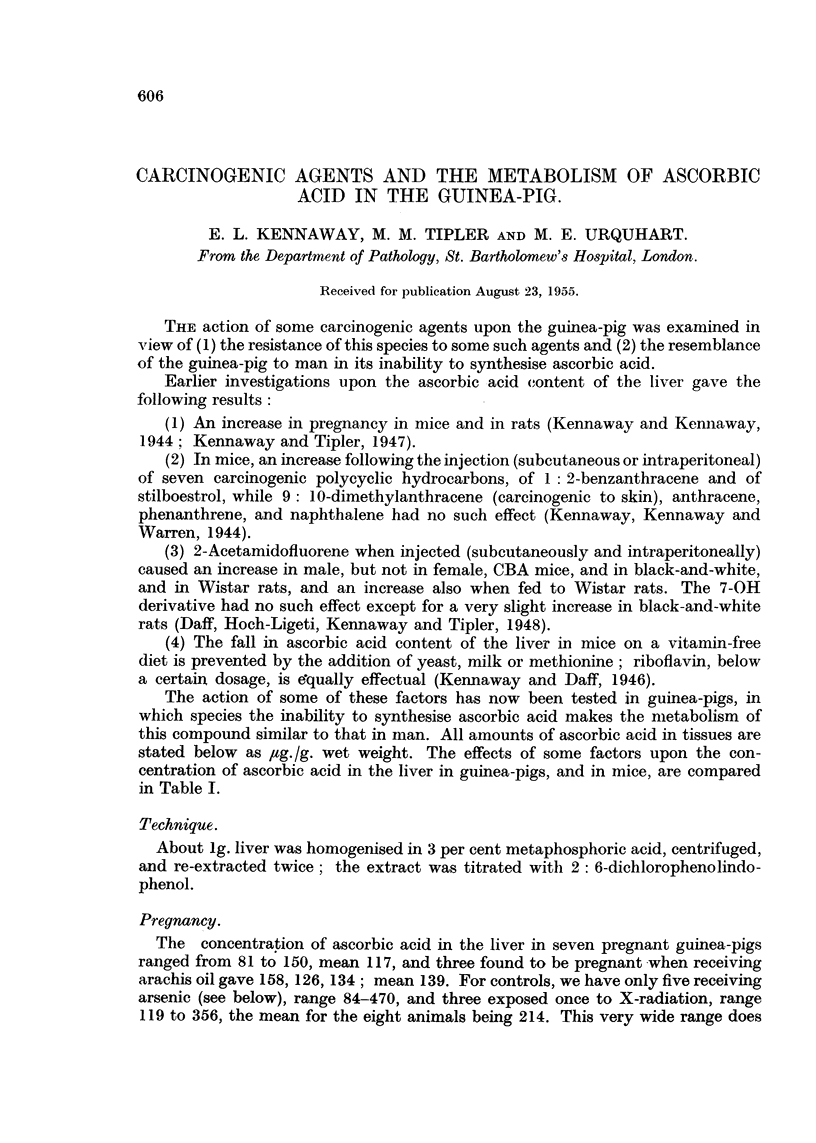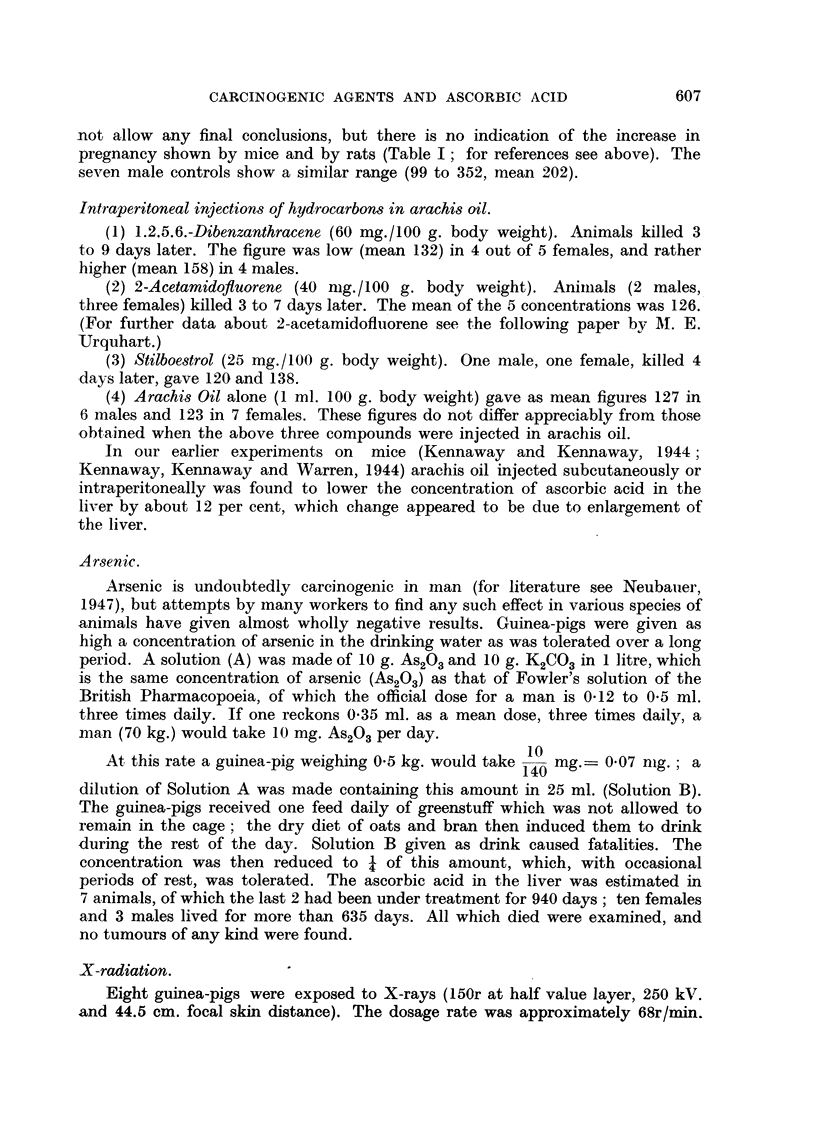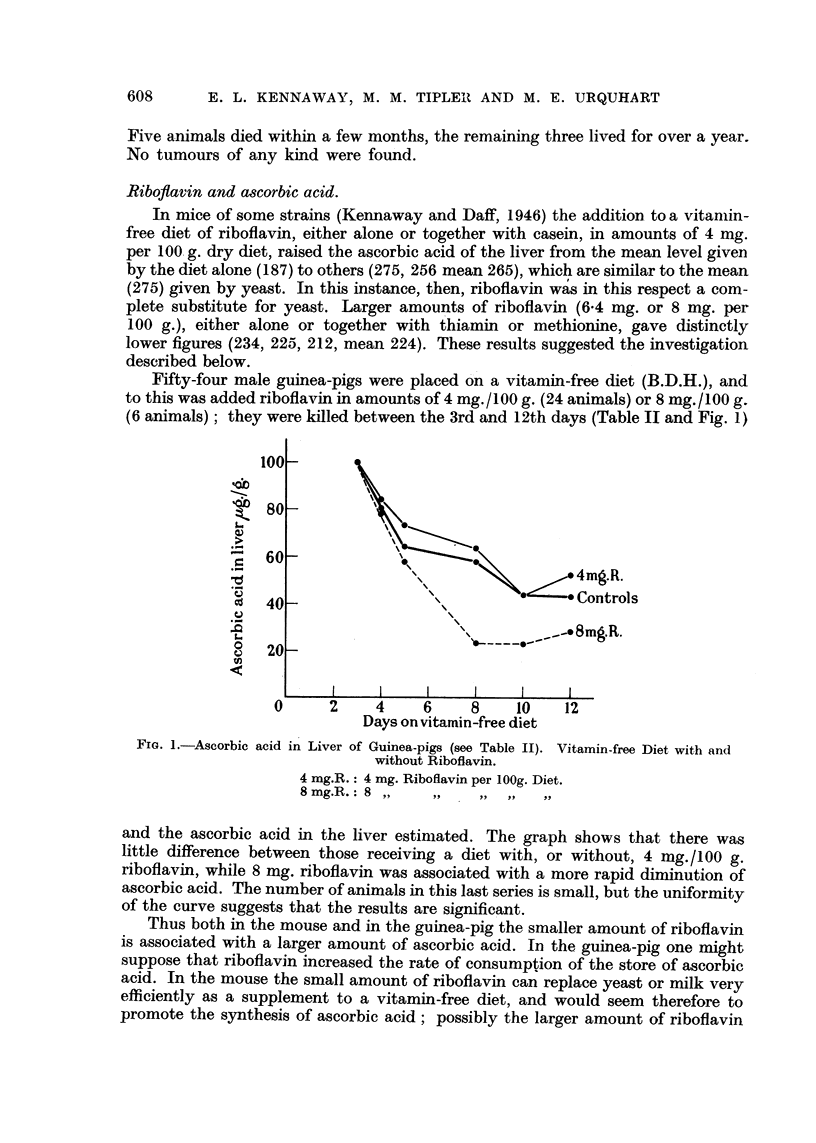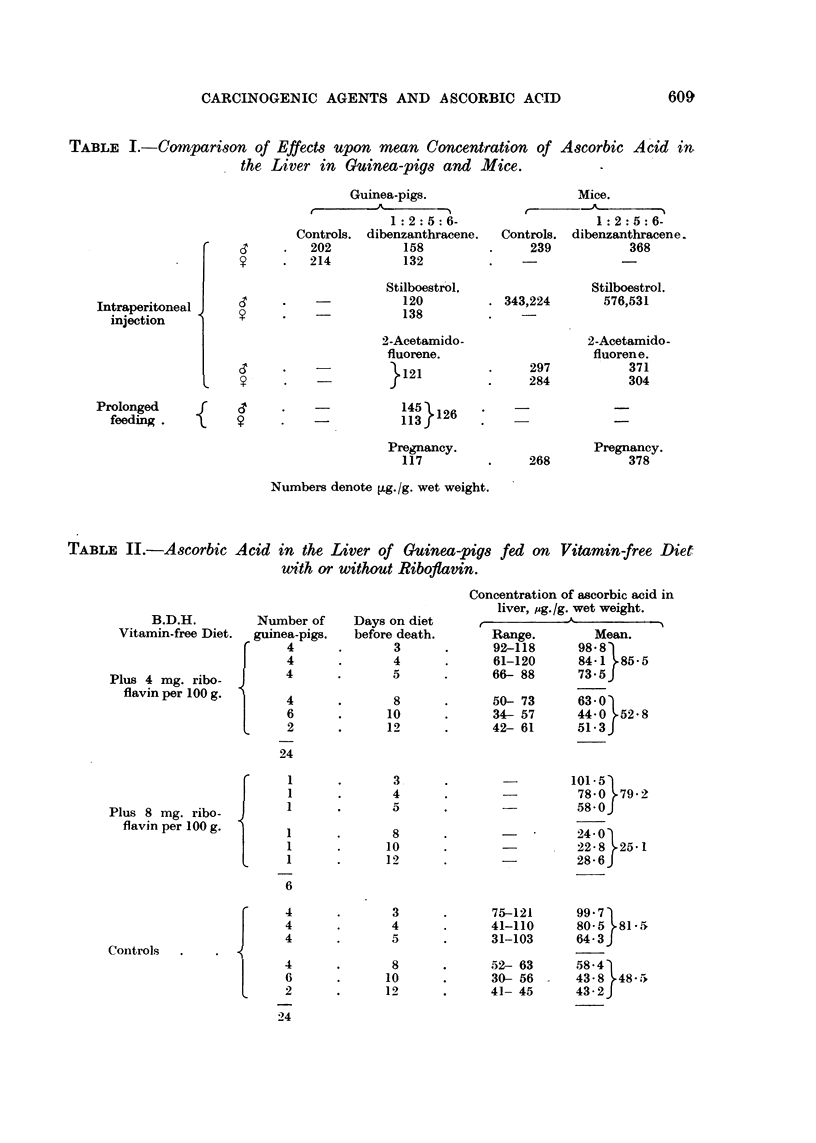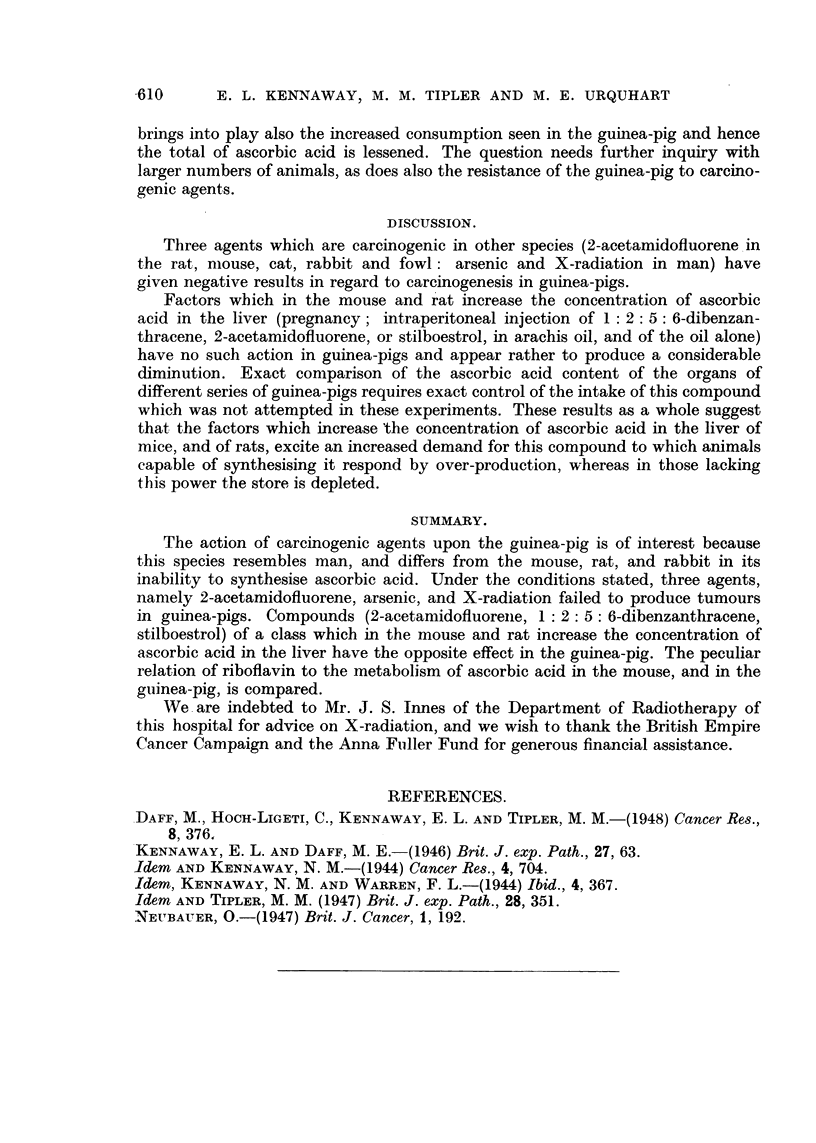# Carcinogenic Agents and the Metabolism of Ascorbic Acid in the Guinea-Pig

**DOI:** 10.1038/bjc.1955.62

**Published:** 1955-12

**Authors:** E. L. Kennaway, M. M. Tipler, M. E. Urquhart


### CARCINOGENIC AGENTS AND THE METABOLISM OF ASCORBIC ACID IN THE GUINEA-PIG.

E. L. KENNAWAY, M. M. TIPLER AND M. E. URQUHART.
*From the Department of Pathology, St. Bartholomew's Hospital, London.*

Received for publication August 23, 1955.

THE action of some carcinogenic agents upon the guinea-pig was examined in view of (1) the resistance of this species to some such agents and (2) the resemblance of the guinea-pig to man in its inability to synthesise ascorbic acid.

Earlier investigations upon the ascorbic acid content of the liver gave the following results :

(1) An increase in pregnancy in mice and in rats (Kennaway and Kennaway, 1944 ; Kennaway and Tipler, 1947).

(2) In mice, an increase following the injection (subcutaneous or intraperitoneal) of seven carcinogenic polycyclic hydrocarbons, of 1 : 2-benzanthracene and of stilboestrol, while 9 : 10-dimethylanthracene (carcinogenic to skin), anthracene, phenanthrene, and naphthalene had no such effect (Kennaway, Kennaway and Warren, 1944).

(3) 2-Acetamidofluorene when injected (subcutaneously and intraperitoneally) caused an increase in male, but not in female, CBA mice, and in black-and-white, and in Wistar rats, and an increase also when fed to Wistar rats. The 7-OH derivative had no such effect except for a very slight increase in black-and-white rats (Daff, Hoch-Ligeti, Kennaway and Tipler, 1948).

(4) The fall in ascorbic acid content of the liver in mice on a vitamin-free diet is prevented by the addition of yeast, milk or methionine ; riboflavin, below a certain dosage, is equally effectual (Kennaway and Daff, 1946).

The action of some of these factors has now been tested in guinea-pigs, in which species the inability to synthesise ascorbic acid makes the metabolism of this compound similar to that in man. All amounts of ascorbic acid in tissues are stated below as $\mu$g./g. wet weight. The effects of some factors upon the concentration of ascorbic acid in the liver in guinea-pigs, and in mice, are compared in Table I.

*Technique.*

About 1g. liver was homogenised in 3 per cent metaphosphoric acid, centrifuged, and re-extracted twice ; the extract was titrated with 2 : 6-dichlorophenolindophenol.

*Pregnancy.*

The concentration of ascorbic acid in the liver in seven pregnant guinea-pigs ranged from 81 to 150, mean 117, and three found to be pregnant when receiving arachis oil gave 158, 126, 134 ; mean 139. For controls, we have only five receiving arsenic (see below), range 84–470, and three exposed once to X-radiation, range 119 to 356, the mean for the eight animals being 214. This very wide range does not allow any final conclusions, but there is no indication of the increase in pregnancy shown by mice and by rats (Table I; for references see above). The seven male controls show a similar range (99 to 352, mean 202).

*Intraperitoneal injections of hydrocarbons in arachis oil.*

(1) 1.2.5.6.-*Dibenzanthracene* (60 mg./100 g. body weight). Animals killed 3 to 9 days later. The figure was low (mean 132) in 4 out of 5 females, and rather higher (mean 158) in 4 males.

(2) 2-*Acetamidofluorene* (40 mg./100 g. body weight). Animals (2 males, three females) killed 3 to 7 days later. The mean of the 5 concentrations was 126. (For further data about 2-acetamidofluorene see the following paper by M. E. Urquhart.)

(3) *Stilboestrol* (25 mg./100 g. body weight). One male, one female, killed 4 days later, gave 120 and 138.

(4) *Arachis Oil* alone (1 ml. 100 g. body weight) gave as mean figures 127 in 6 males and 123 in 7 females. These figures do not differ appreciably from those obtained when the above three compounds were injected in arachis oil.

In our earlier experiments on mice (Kennaway and Kennaway, 1944; Kennaway, Kennaway and Warren, 1944) arachis oil injected subcutaneously or intraperitoneally was found to lower the concentration of ascorbic acid in the liver by about 12 per cent, which change appeared to be due to enlargement of the liver.

*Arsenic.*

Arsenic is undoubtedly carcinogenic in man (for literature see Neubauer, 1947), but attempts by many workers to find any such effect in various species of animals have given almost wholly negative results. Guinea-pigs were given as high a concentration of arsenic in the drinking water as was tolerated over a long period. A solution (A) was made of 10 g. $As_2O_3$ and 10 g. $K_2CO_3$ in 1 litre, which is the same concentration of arsenic ($As_2O_3$) as that of Fowler's solution of the British Pharmacopoeia, of which the official dose for a man is 0·12 to 0·5 ml. three times daily. If one reckons 0·35 ml. as a mean dose, three times daily, a man (70 kg.) would take 10 mg. $As_2O_3$ per day.

At this rate a guinea-pig weighing 0·5 kg. would take $\frac{10}{140}$ mg. = 0·07 mg.; a dilution of Solution A was made containing this amount in 25 ml. (Solution B). The guinea-pigs received one feed daily of greenstuff which was not allowed to remain in the cage; the dry diet of oats and bran then induced them to drink during the rest of the day. Solution B given as drink caused fatalities. The concentration was then reduced to $\frac{1}{4}$ of this amount, which, with occasional periods of rest, was tolerated. The ascorbic acid in the liver was estimated in 7 animals, of which the last 2 had been under treatment for 940 days; ten females and 3 males lived for more than 635 days. All which died were examined, and no tumours of any kind were found.

*X-radiation.*

Eight guinea-pigs were exposed to X-rays (150r at half value layer, 250 kV. and 44.5 cm. focal skin distance). The dosage rate was approximately 68r/min.

Five animals died within a few months, the remaining three lived for over a year. No tumours of any kind were found.

*Riboflavin and ascorbic acid.*

In mice of some strains (Kennaway and Daff, 1946) the addition to a vitamin-free diet of riboflavin, either alone or together with casein, in amounts of 4 mg. per 100 g. dry diet, raised the ascorbic acid of the liver from the mean level given by the diet alone (187) to others (275, 256 mean 265), which are similar to the mean (275) given by yeast. In this instance, then, riboflavin was in this respect a complete substitute for yeast. Larger amounts of riboflavin (6·4 mg. or 8 mg. per 100 g.), either alone or together with thiamin or methionine, gave distinctly lower figures (234, 225, 212, mean 224). These results suggested the investigation described below.

Fifty-four male guinea-pigs were placed on a vitamin-free diet (B.D.H.), and to this was added riboflavin in amounts of 4 mg./100 g. (24 animals) or 8 mg./100 g. (6 animals); they were killed between the 3rd and 12th days (Table II and Fig. 1)

FIG. 1.—Ascorbic acid in Liver of Guinea-pigs (see Table II). Vitamin-free Diet with and without Riboflavin.

4 mg.R.: 4 mg. Riboflavin per 100g. Diet.
8 mg.R.: 8 ,, ,, ,, ,, ,, and the ascorbic acid in the liver estimated. The graph shows that there was little difference between those receiving a diet with, or without, 4 mg./100 g. riboflavin, while 8 mg. riboflavin was associated with a more rapid diminution of ascorbic acid. The number of animals in this last series is small, but the uniformity of the curve suggests that the results are significant.

Thus both in the mouse and in the guinea-pig the smaller amount of riboflavin is associated with a larger amount of ascorbic acid. In the guinea-pig one might suppose that riboflavin increased the rate of consumption of the store of ascorbic acid. In the mouse the small amount of riboflavin can replace yeast or milk very efficiently as a supplement to a vitamin-free diet, and would seem therefore to promote the synthesis of ascorbic acid; possibly the larger amount of riboflavin

TABLE I.—*Comparison of Effects upon mean Concentration of Ascorbic Acid in the Liver in Guinea-pigs and Mice.*

| | | Guinea-pigs. | | Mice. | |
|---|---|---|---|---|---|
| | | Controls. | 1 : 2 : 5 : 6-dibenzanthracene. | Controls. | 1 : 2 : 5 : 6-dibenzanthracene. |
| Intraperitoneal injection | ♂ | 202 | 158 | 239 | 368 |
| | ♀ | 214 | 132 | — | — |
| | | | Stilboestrol. | | Stilboestrol. |
| | ♂ | — | 120 | 343,224 | 576,531 |
| | ♀ | — | 138 | — | |
| | | | 2-Acetamido-fluorene. | | 2-Acetamido-fluorene. |
| | ♂ | — | } 121 | 297 | 371 |
| | ♀ | — | | 284 | 304 |
| Prolonged feeding | ♂ | — | 145 } 126 | — | — |
| | ♀ | — | 113 | — | — |
| | | | Pregnancy. | | Pregnancy. |
| | | | 117 | 268 | 378 |

Numbers denote µg./g. wet weight.

TABLE II.—*Ascorbic Acid in the Liver of Guinea-pigs fed on Vitamin-free Diet with or without Riboflavin.*

| B.D.H. Vitamin-free Diet. | Number of guinea-pigs. | Days on diet before death. | Concentration of ascorbic acid in liver, µg./g. wet weight. Range. | Mean. | |
|---|---|---|---|---|---|
| Plus 4 mg. riboflavin per 100 g. | 4 | 3 | 92–118 | 98·8 | 85·5 |
| | 4 | 4 | 61–120 | 84·1 | |
| | 4 | 5 | 66– 88 | 73·5 | |
| | 4 | 8 | 50– 73 | 63·0 | 52·8 |
| | 6 | 10 | 34– 57 | 44·0 | |
| | 2 | 12 | 42– 61 | 51·3 | |
| | 24 | | | | |
| Plus 8 mg. riboflavin per 100 g. | 1 | 3 | — | 101·5 | 79·2 |
| | 1 | 4 | — | 78·0 | |
| | 1 | 5 | — | 58·0 | |
| | 1 | 8 | — | 24·0 | 25·1 |
| | 1 | 10 | — | 22·8 | |
| | 1 | 12 | — | 28·6 | |
| | 6 | | | | |
| Controls | 4 | 3 | 75–121 | 99·7 | 81·5 |
| | 4 | 4 | 41–110 | 80·5 | |
| | 4 | 5 | 31–103 | 64·3 | |
| | 4 | 8 | 52– 63 | 58·4 | 48·5 |
| | 6 | 10 | 30– 56 | 43·8 | |
| | 2 | 12 | 41– 45 | 43·2 | |
| | 24 | | | | |

brings into play also the increased consumption seen in the guinea-pig and hence the total of ascorbic acid is lessened. The question needs further inquiry with larger numbers of animals, as does also the resistance of the guinea-pig to carcinogenic agents.

### DISCUSSION.

Three agents which are carcinogenic in other species (2-acetamidofluorene in the rat, mouse, cat, rabbit and fowl: arsenic and X-radiation in man) have given negative results in regard to carcinogenesis in guinea-pigs.

Factors which in the mouse and rat increase the concentration of ascorbic acid in the liver (pregnancy; intraperitoneal injection of 1 : 2 : 5 : 6-dibenzanthracene, 2-acetamidofluorene, or stilboestrol, in arachis oil, and of the oil alone) have no such action in guinea-pigs and appear rather to produce a considerable diminution. Exact comparison of the ascorbic acid content of the organs of different series of guinea-pigs requires exact control of the intake of this compound which was not attempted in these experiments. These results as a whole suggest that the factors which increase the concentration of ascorbic acid in the liver of mice, and of rats, excite an increased demand for this compound to which animals capable of synthesising it respond by over-production, whereas in those lacking this power the store is depleted.

### SUMMARY.

The action of carcinogenic agents upon the guinea-pig is of interest because this species resembles man, and differs from the mouse, rat, and rabbit in its inability to synthesise ascorbic acid. Under the conditions stated, three agents, namely 2-acetamidofluorene, arsenic, and X-radiation failed to produce tumours in guinea-pigs. Compounds (2-acetamidofluorene, 1 : 2 : 5 : 6-dibenzanthracene, stilboestrol) of a class which in the mouse and rat increase the concentration of ascorbic acid in the liver have the opposite effect in the guinea-pig. The peculiar relation of riboflavin to the metabolism of ascorbic acid in the mouse, and in the guinea-pig, is compared.

We are indebted to Mr. J. S. Innes of the Department of Radiotherapy of this hospital for advice on X-radiation, and we wish to thank the British Empire Cancer Campaign and the Anna Fuller Fund for generous financial assistance.

### REFERENCES.

Daff, M., Hoch-Ligeti, C., Kennaway, E. L. and Tipler, M. M.—(1948) *Cancer Res.*, **8**, 376.

Kennaway, E. L. and Daff, M. E.—(1946) *Brit. J. exp. Path.*, **27**, 63.

*Idem* and Kennaway, N. M.—(1944) *Cancer Res.*, **4**, 704.

*Idem*, Kennaway, N. M. and Warren, F. L.—(1944) *Ibid.*, **4**, 367.

*Idem* and Tipler, M. M. (1947) *Brit. J. exp. Path.*, **28**, 351.

Neubauer, O.—(1947) *Brit. J. Cancer*, **1**, 192.